# Giant endobronchial hamartoma resected by fiberoptic bronchoscopy electrosurgical snaring

**DOI:** 10.1186/1749-8090-6-97

**Published:** 2011-08-14

**Authors:** Baldassare Mondello, Salvatore Lentini, Carmelo Buda, Francesco Monaco, Dario Familiari, Michele Sibilio, Annunziata La Rocca, Pietro Barresi, Vittorio Cavallari, Maurizio Monaco, Mario Barone

**Affiliations:** 1 Thoracic Surgery Unit, Cardiovascular and Thoracic Department, Policlinic University Hospital, University of Messina, Italy; 2 Department of human pathology, Policlinic University Hospital, University of Messina, Italy

**Keywords:** tumor (lung), Pathology (lung), Hamartoma, Lung cancer, Imaging

## Abstract

Less than 1% of lung neoplasms are represented by benign tumors. Among these, hamartomas are the most common with an incidence between 0.025% and 0.32%. In relation to the localization, hamartomas are divided into intraparenchymal and endobronchial.

Clinical manifestation of an endobronchial hamartoma (EH) results from tracheobronchial obstruction or bleeding. Usually, EH localizes in large diameter bronchus. Endoscopic removal is usually recommended. Bronchotomy or parenchimal resection through thoracotomy should be reserved only for cases where the hamatoma cannot be approached through endoscopy, or when irreversible lung functional impairment occurred after prolonged airflow obstruction. Generally, when endoscopic approach is used, this is through rigid bronchoscopy, laser photocoagulation or mechanical resection. Here we present a giant EH occasionally diagnosed and treated by fiberoptic bronchoscopy electrosurgical snaring.

## Introduction

Most tumors of the tracheobronchial tree are malignant [1,2]. Benign lung tumors represent less than 1%, and among these, hamartomas, with an incidence between 0.025% and 0.32%, are the most common [3]. In relation to the localization, hamartomas are divided into intraparenchymal, generally asymptomatic and with a radiological coin lesion appearance [4], and endobronchial, clinically manifesting as a result of tracheobronchial obstruction [5].

From a previous paper reviewing a total of 215 cases of hamartoma reported in the literature, the endobronchial location was found in only 1.4% of cases [6]. In contrast, other studies found an incidence of endobronchial location in 10 and 20% of all pulmonary hamartomas [7,8]. The endobronchial hamartomas (EH) usually localize in large diameter bronchus [2]. Since these tumors are benign, endoscopic removal is usually recommended, reserving lung resection to cases of longstanding bronchial obstruction with infection and irreversible lung injury [9].

We report the case of a giant hamartoma of the left main bronchus, diagnosed and removed by fiberoptic bronchoscopy electrosurgical snaring.

## Case report

**Figure 1:**
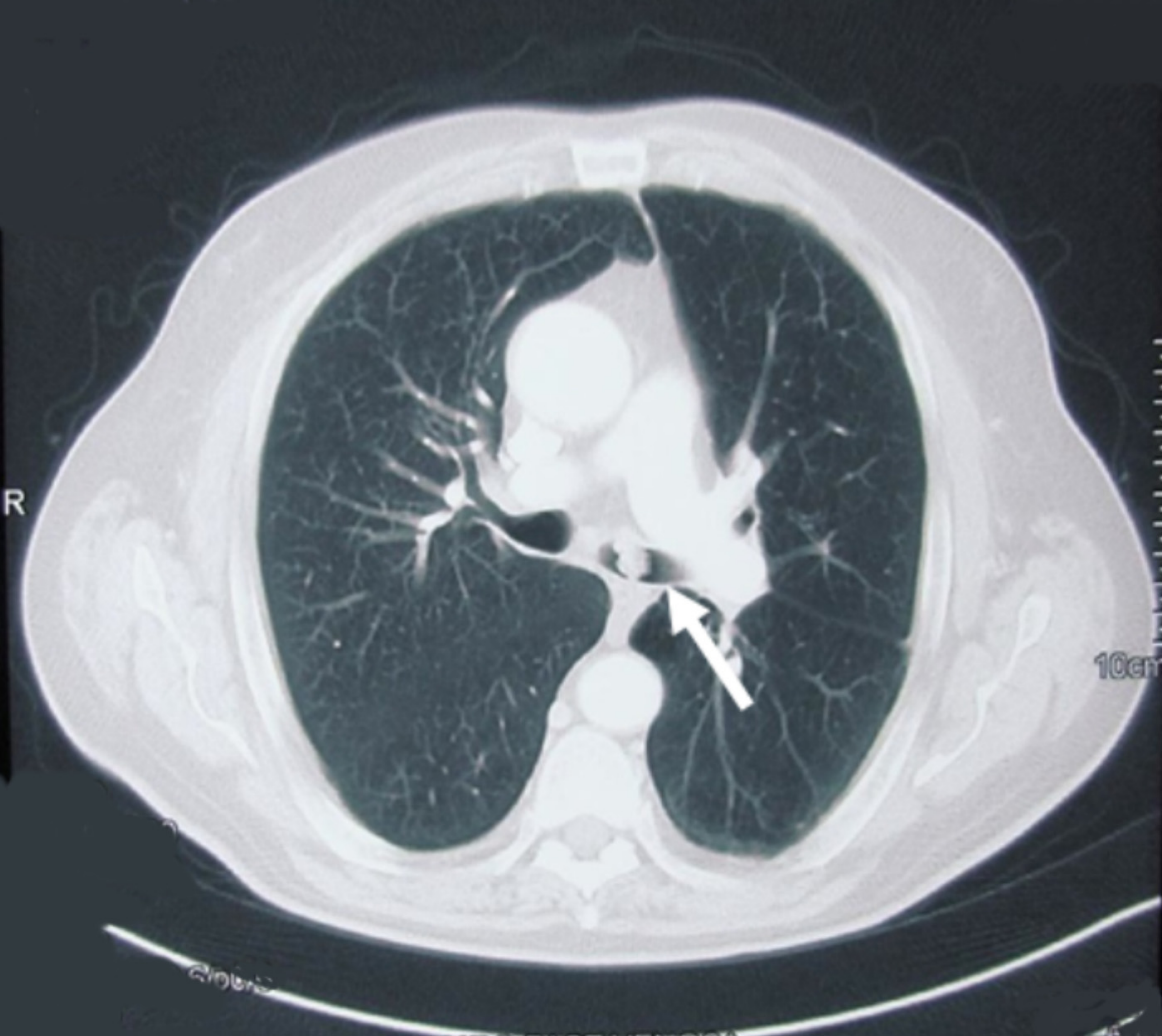
**CT scan demonstrating a vegetating neoplasm of the left main bronchus (white arrow) without signs of extrabronchial infiltration**.

**Figure 2:**
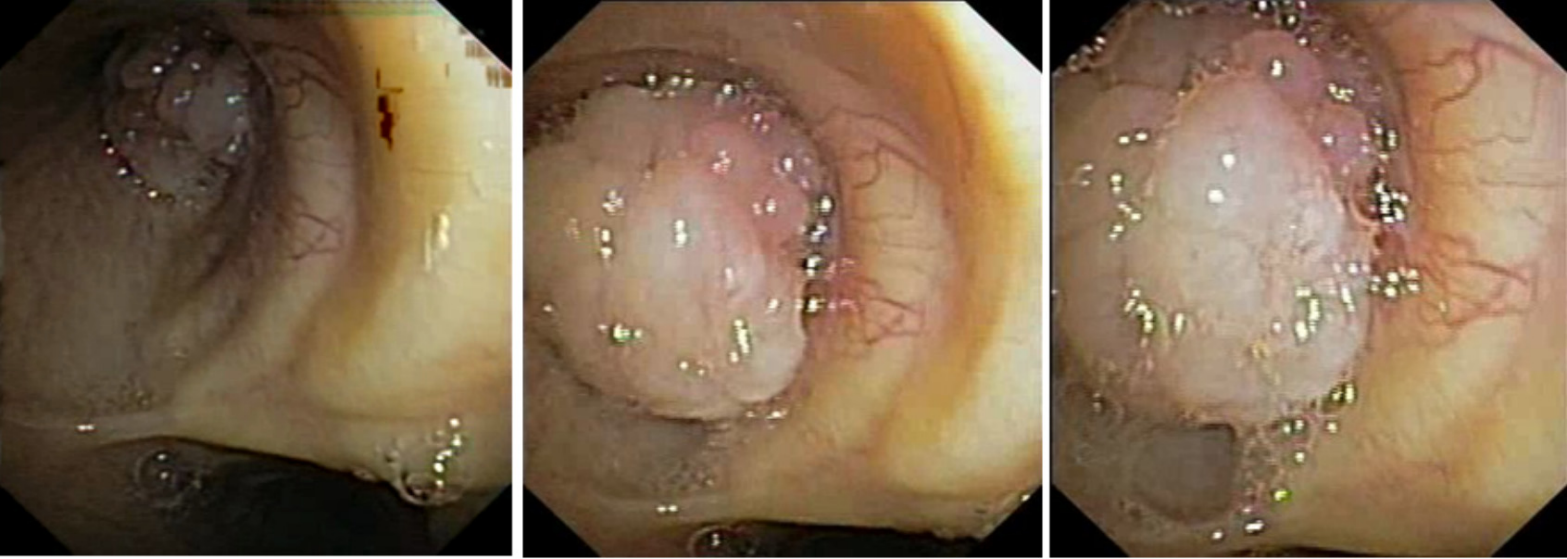
**Bronchoscopy detects a vegetating lesion, moving during the act of breathing, nearly occluding the lumen of the left main bronchus**.

**Figure 3:**
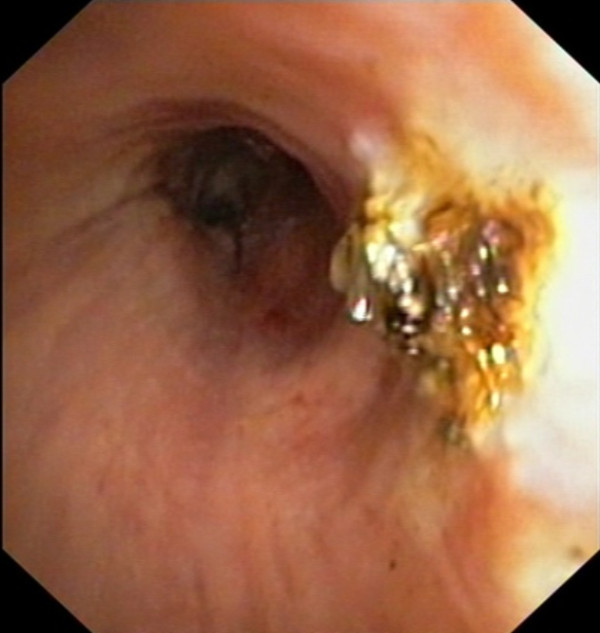
**Result at the end of the procedure: Macroscopically complete lesion resection by fiberoptic bronchoscopy electrosurgical snaring**.

**Figure 4:**
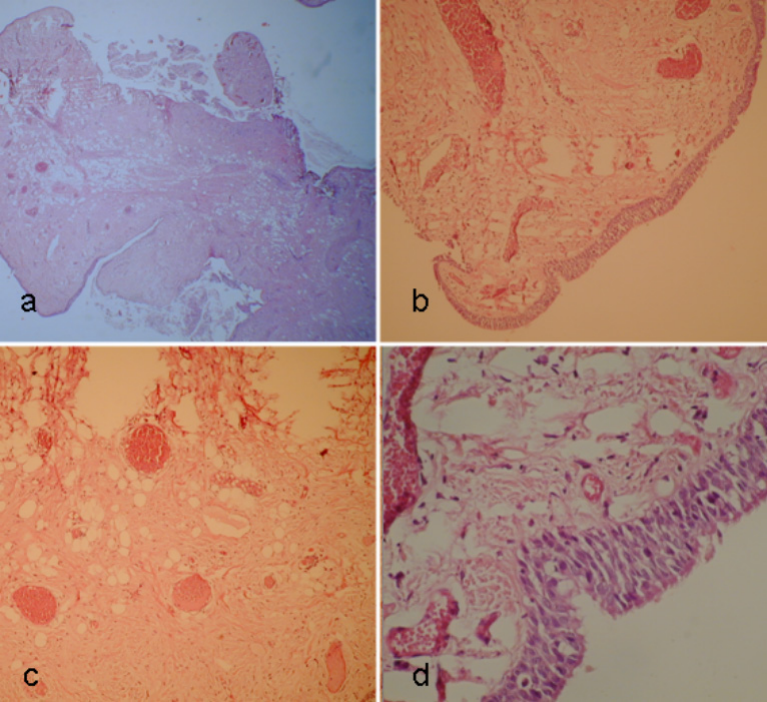
**Histological aspects:** a) At low magnification: absence of ulcerations. b) Fibro-vascular architecture. c) group of adipocytes. d) epithelial lining.

**Figure 5:**
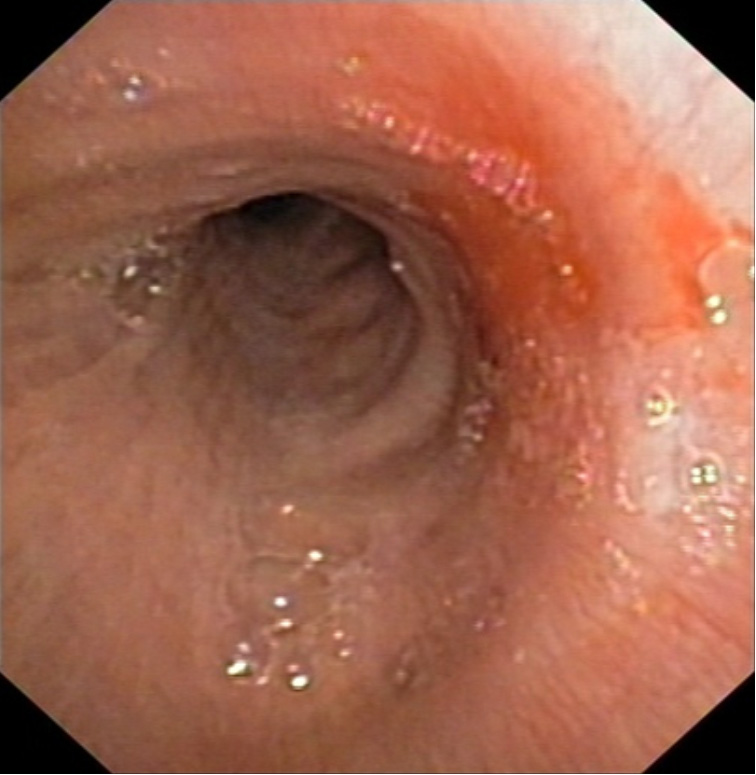
**Postoperative endoscopic control at 30 days showing good epithelialization of the mucosa**.

An asymptomatic 65 year old man, previously treated by rectum resection for adenocarcinoma, during follow-up examination for his neoplastic disease underwent chest CT scan that documented a vegetating lesion of the left main bronchus with absence of extra-bronchial infiltration (Figure 1). The patient underwent diagnostic bronchoscopy that confirmed the presence in the left main bronchus, at about 2.5 cm from the carina, of a vegetating, pedunculated lesion, mobile during breathing and nearly occluding the bronchial lumen (Figure 2). However, despite the large tumor size, air entry into the left lung was allowed probably during the tumor movements inside the bronchial lumen. Cyto-histological samples were suggestive of a hamartoma. Endoscopic resection of the lesion was then performed using fiberoptic bronchoscopy electrosurgical snaring, obtaining macroscopic total removal (Figure 3). The definitive histological diagnosis returned as "bronchial hamartoma with predominant fibrovascular structure" (Figure 4). Postoperative endoscopic control at 10 and 30 days showed good re-epithelialization of the bronchial mucosa (Figure 5). Endoscopic control at the 6 month follow-up showed no recurrence.

## Discussion

The pulmonary hamartoma is a rare benign tumor, originating from the bronchial primitive mesenchymal tissue, which can differentiate into various mature mesenchymal components [8]. In fact, the hamartoma, either intra-parenchymal or endobronchial, generally includes cartilage, bone, fat and muscle tissues [5]. Usually, EH has a higher fat content than intraparenchymal hamarthoma [10]. Generally, the cartilaginous component prevails over others, even though forms with predominantly fatty or bone components have been described as well [1].

EH is frequently asymptomatic, at least in the preocclusive early stage [5]. When present, symptoms are secondary to tracheobronchial obstruction, resulting in recurrent pneumonias, and include fever, cough, hemoptysis, purulent sputum, dyspnea and pain [5,6,11,12]. Sometimes, recurrent pneumonias secondary to bronchial obstruction may irreversibly damage the lung or part of it [13].

On CT scan, the EH appears as an endobronchial mass with or without signs of obstructive pneumonia or atelectasia [2]. CT scan is of considerable diagnostic aid in cases of EH with high fat content [14]. Stey *et al.* considered highly indicative the presence on CT scan of a mass at high fat density without contrast uptake [1].

At bronchoscopic examination, the EH appears as a polypoid or pedunculated neoplasm, well-circumscribed, with a smooth and yellowish surface, without signs of submucosal infiltration [1,2]. Biopsies are necessary for the differential diagnosis from other benign neoplasms and from carcinoid [1]. Histology would usually detect the coexistence of connective, epithelial, bone, muscle, fat and cartilage tissues, the latter usually in high prevalence [1,9,12].

The traditional treatment has been by thoracotomy with broncotomy or lung resection (12). However, since this is a benign neoplasm, endoscopic treatment is now widely recommended as the first line approach [1,2,4], also considering that malignant degeneration is extremely rare and the recurrence rate is low [2,5,6]. Generally, the endoscopic approach is through rigid bronchoscopy, laser photocoagulation or mechanical resection [15-18].

Laser treatment through rigid bronchoscopy is considered the gold standard treatment for symptomatic patients with bulky masses on radiological examination [5]. However, in selected cases, the use of electrocautery through flexible bronchoscopy may prove just as simple and effective [4,18]. Endoscopic electrosurgical snaring is widely used in gastroenterology [4], while its use in tracheobronchial endoscopy is rare. It is still not fully known the depth of electrocauterization [4]. Possible complications may include bleeding, perforation and burning lesions on the tracheobronchial tree [19].

The traditional surgical treatment (thoracotomy and bronchotomy) is currently indicated only in cases where the EH cannot be approached through endoscopy, or when lung resection is indicated due to irreversible parenchymal damage from longstanding airway obstruction [9,20].

## Conclusions

The EH is a rare benign tumor that can cause bleeding or obstruction of the tracheobronchial tree.

For these reasons, treatment should be performed even in asymptomatic patients. The choice of treatment should consider the location and extent of the tumor. Surgical therapy, by bronchotomy or resection, should be reserved only for cases where the hamatoma cannot be approached through endoscopy, or when irreversible lung functional impairment occurred after prolonged airflow obstruction. In all other cases, in consideration of the benign nature of the tumor, the gold standard treatment is endoscopic laser resection. Fiberoptic bronchoscopy electrosurgical snaring may represent an alternative approach in selected cases.

## Consent

Written informed consent was obtained from patients for publication of this report and accompanying images. A copy of the written consent is available for review by the Editor in chief of this journal.

## Competing interests

The authors declare that they have no competing interests.

## Authors' contributions

All authors: 1. have made substantial contributions to conception and design, or acquisition of data, or analysis and interpretation of data; 2. have been involved in drafting the manuscript or revisiting it critically for important intellectual content; 3. have given final approval of the version to be published.
